# Origins and Domestication of Cultivated Banana Inferred from Chloroplast and Nuclear Genes

**DOI:** 10.1371/journal.pone.0080502

**Published:** 2013-11-18

**Authors:** Lin-Feng Li, Hua-Ying Wang, Cui Zhang, Xin-Feng Wang, Feng-Xue Shi, Wen-Na Chen, Xue-Jun Ge

**Affiliations:** 1 Key Laboratory of Molecular Epigenetics of Ministry of Education, Northeast Normal University, Changchun, China; 2 Key Laboratory of Plant Resources Conservation and Sustainable Utilization, South China Botanical Garden, the Chinese Academy of Sciences, Guangzhou, China; Wuhan Botanical Garden, Chinese Academy of Sciences, Wuhan, China

## Abstract

**Background:**

Cultivated bananas are large, vegetatively-propagated members of the genus *Musa*. More than 1,000 cultivars are grown worldwide and they are major economic and food resources in numerous developing countries. It has been suggested that cultivated bananas originated from the islands of Southeast Asia (ISEA) and have been developed through complex geodomestication pathways. However, the maternal and parental donors of most cultivars are unknown, and the pattern of nucleotide diversity in domesticated banana has not been fully resolved.

**Methodology/Principal Findings:**

We studied the genetics of 16 cultivated and 18 wild *Musa* accessions using two single-copy nuclear (granule-bound starch synthase I, *GBSS I*, also known as *Waxy*, and alcohol dehydrogenase 1, *Adh*1) and two chloroplast (maturase K, *mat*K, and the *trnL-F* gene cluster) genes. The results of phylogenetic analyses showed that all A-genome haplotypes of cultivated bananas were grouped together with those of ISEA subspecies of *M. acuminata* (A-genome). Similarly, the B- and S-genome haplotypes of cultivated bananas clustered with the wild species *M. balbisiana* (B-genome) and *M. schizocarpa* (S-genome), respectively. Notably, it has been shown that distinct haplotypes of each cultivar (A-genome group) were nested together to different ISEA subspecies *M. acuminata*. Analyses of nucleotide polymorphism in the *Waxy* and *Adh1* genes revealed that, in comparison to the wild relatives, cultivated banana exhibited slightly lower nucleotide diversity both across all sites and specifically at silent sites. However, dramatically reduced nucleotide diversity was found at nonsynonymous sites for cultivated bananas.

**Conclusions/Significance:**

Our study not only confirmed the origin of cultivated banana as arising from multiple intra- and inter-specific hybridization events, but also showed that cultivated banana may have not suffered a severe genetic bottleneck during the domestication process. Importantly, our findings suggested that multiple maternal origins and a reduction in nucleotide diversity at nonsynonymous sites are general attributes of cultivated bananas.

## Introduction

Cultivated bananas are the fourth most important crop in developing countries, and it has been proposed that they derive from the domestication of genus *Musa* [1], [2]. Previous archaeological and linguistic studies have indicated that cultivated banana was initially domesticated by farmers in Southeast Asia about 7,000 years ago, and subsequently introduced into other regions of the world by transmigrants or travelers [1], [2], [3]. Nowadays, more than one thousand landraces of domesticated banana are cultivated in the tropical and subtropical regions of the world [1]. To gain a better understanding of the origin and domestication of cultivated banana, a series of studies using morphological and molecular methods has focused on the systematics and classification of members of the genus *Musa*. For example, Cheesman [4] divided this genus into sections *Eumusa* (*x* = 11), *Rhodochlamys* (*x* = 11), *Callimusa* (*x* = 10) and *Australimusa* (*x* = 10) based on morphological traits and basic chromosome number. This classification system was modified by Simmonds [5], [6] and Argent [7], who included a new section *Ingentimusa* (*x* = 7) due to the different basic chromosome number. There have been extensive discussions about the identities of the progenitors of domesticated banana; *M. acuminata* and *M. balbisiana* have been proposed as the wild parents of modern bananas [6]. This hypothesis was subsequently confirmed by genetic studies of the genus *Musa* which indicated that at least four wild species, *M. acuminata* (donor of the A genome), *M. balbisiana* (donor of the B genome), *M. schizocarpa* (donor of the S genome) and *M. textilis* (donor of the T genome), have contributed to the gene pools of domesticated bananas [1], [8], [9]. At present, these four wild relatives are still widespread in the tropical and subtropical regions of Asia.

The species *M. acuminata* (section *Eumusa*) is widely distributed in the tropical and subtropical regions of Asia and at least nine subspecies have been identified: *banksii*, *burmannica*, *burmannicoides*, *errans*, *malaccensis*, *microcarpa*, *siamea*, *truncata* and *zebrina* [10], [11], [12]. Although no subspecies categories have been designated in *M. balbisiana* (section *Musa*), it also exhibits wide variation in morphological characters and is distributed across the tropical and subtropical regions of Asia. In contrast, however, the other two wild progenitors, *M. schizocarpa* (section *Musa*) and *M. textilis* (section *Callimusa*), are endemic to Papua New Guinea and Philippines respectively, and show no obvious morphological diversification [13]. Cultivated bananas differ from their wild relatives in being seedless and parthenocarpic; that is, the fruit develops without seed development or pollination and fertilization [1]. Although cultivated bananas reproduce through vegetative propagation, they exhibit a high level of morphological diversification in fruit size, shape and color. To provide a framework for banana classification, Simmonds and Shepherd [3] divided cultivated bananas into genotypes AA, AB, AAA, AAB and ABB on the basis of qualitative morphological descriptors and genome composition. This system provides a clear and coherent classification for cultivated banana and has, therefore, been widely accepted.

Although domesticated bananas are of socioeconomic importance, genetic studies on them have been limited due to the existence of polyploidy and parthenocarpy and to the difficulties inherent in sample collection. To elucidate the systematic relationships and genetic diversity of *Musa* germplasm, several studies have evaluated the genomic constitution of cultivated banana and its wild relatives using restriction fragment length polymorphism (RFLP), amplified fragment length polymorphism (AFLP) and simple sequence repeat (SSR) markers [2], [14], [15], [16], [17], [18]. These studies have revealed that cultivated bananas originated from the genus *Musa* through complex geodomestication pathways. To date, although the major stages in banana domestication have been clarified, the maternal and parental donors of most cultivars are unknown, and the pattern of nucleotide diversity in domesticated banana has not yet been fully resolved.

In this study, to gain a better understanding of the origin and domestication of cultivated banana, we performed genetic analyses of 16 banana cultivars and 18 wild *Musa* accessions using two single copy nuclear genes: granule-bound starch synthase I (*GBSS* I or *Waxy*) and alcohol dehydrogenase 1 (*Adh*1). The product of *Waxy* is a key enzyme in amylose synthesis. It has been shown that mutations in *Waxy* (null alleles) can result in a reduction in amylose content [19]. *Adh*1 is a member of the alcohol dehydrogenase gene family whose products catalyze the NAD^+^-dependent oxidation of alcohols. The *Waxy* and *Adh*1 genes are the plant nuclear regions that have been most intensively investigated in studies on molecular phylogeny and population genetics [20], [21], [22], [23], [24], [25]. For example, Guzman et al. [25] employed the *Waxy* gene to evaluate the phylogenetic relationships and origins of different types of wheat and revealed that Iberian spelt has a different origin from other spelts. In addition, Yoshida et al. [22] examined the nucleotide diversity of the *Adh1* gene in *Oryza rufipogon* and found that this gene showed relatively low genetic diversity in comparison with other loci investigated in this species. Taken together, these previous studies have demonstrated that *Waxy* and *Adh1* are ideal nuclear loci for use in investigating the molecular phylogenetic and nucleotide diversity of domesticated plants. To further infer the paternal and maternal donors of the cultivars investigated in the present study, we also surveyed the sequences of two chloroplast fragments maturase K (*mat*K) and *trnL-F*. Our aims are to (i) reveal the origins of, and the domestication process that gave rise to, cultivated bananas and (ii) assess the genetic diversity of domesticated bananas and their wild relatives.

## Materials and Methods

### Plant Material

The *Musa* accessions used in this study were obtained from the Biodiversity International Transit Centre (ITC) and our own collections. As shown in Table 1, 16 cultivated accessions were sampled from 12 countries; they contain the six major genotypes (AA, AB, AS, AAA, AAB and ABB). For each genotype, multiple accessions were collected to represent different subgroups and to cover their geographical ranges. Similarly, the wild *Musa* accessions were selected to cover their natural geographic distributions and presented most of their varieties or subspecies.

**Table 1:** Details of the *Musa* accessions used in this study.

Sample number	Code	Name	Section	Species/Group	Sub-species/Sub-group	Country of collection
1	ITC0312	Pisang Jari Buaya	*Musa*	AA cv	Pisang jari buaya	Malaysia, kelatan Thai border
2	ITC0653	Pisang Mas	*Musa*	AA cv	Sucrier	Malaysia
3	-	Gongjiao	*Musa*	AA cv	Gongjiao	Vietnam
4	-	Haigong	*Musa*	AA cv	Gongjiao	China
5	ITC1187	Tomolo	*Musa*	AA cv	Cooking AA	Papua New Guinea (PNG023)
6	ITC1034	Kunnan	*Musa*	AB cv	-	India, Kerala
7	ITC1152	Wompa	*Musa*	AS cv	-	Papua New Guinea (PNG 063)
8	NEU0172	Grande Naine	*Musa*	AAA cv	Cavendish	Guadeloupe
9	ITC0420	Pisang Kayu	*Musa*	AAA cv	Orotav	Indonesia (IDN 098)
10	ITC1481	Gros Michel	*Musa*	AAA cv	Gros Michel	Guadeloupe
11	ITC0084	Mbwazirume	*Musa*	AAA cv	Lujugira/Mutika	Burundi
12	ITC0649	Foconah	*Musa*	AAB cv	Pome/Prata	Cameroon
13	ITC1325	Orishele	*Musa*	AAB cv	Plantain	Nigeria
14	ITC1140	Red Yade	*Musa*	AAB cv	Plantain	Cameroon
15	ITC0843	Pisang Raja Bulu	*Musa*	AAB cv	Pisang raja	Indonesia (IND 093)
16	ITC0659	Namwa Khom	*Musa*	ABB cv	Pisang Awak	Thailand (THA 011)
17	ITC0623	Banksii	*Musa*	*acuminata*	*banksii*	Papua New Guinea
18	ITC0253	Borneo	*Musa*	*acuminata*	*microcarpa*	Malaysia, S/E Borneo
19	ITC0249	Calcutta 4	*Musa*	*acuminata*	*burmannicoides*	India
20	ITC1028	Agutay	*Musa*	*acuminata*	*errans*	Philippines
21	ITC0660	Khae (Phrae)	*Musa*	*acuminata*	*siamea*	Thailand (THA 015)
22	ITC1177	Zebrina	*Musa*	*acuminata*	*zebrina*	Indonesia
23	-	Zebrina	*Musa*	*acuminata*	*zebrina*	Indonesia
24	-	malaccensis	*Musa*	*acuminata*	*malaccensis*	-
25	ITC0283	Long Tavoy	*Musa*	*acuminata*	*burmannica*	-
26	-	-	*Musa*	*acuminata*	*burmannica*	China
27	-	-	*Musa*	*acuminata*	-	Burma
28	-	-	*Musa*	*acuminata*	-	China
29	ITC0247	Honduras	*Musa*	*balbisiana*	type 1	Honduras (seeds)
30	NEU0051	Lal Velchi	*Musa*	*balbisiana*	type 3	India
31	ITC1120	Tani	*Musa*	*balbisiana*	-	-
32	ITC1063	Pisang Klutuk Wulung	*Musa*	*balbisiana*	type 4	Indonesia (IDN 056)
33	ITC1156	Pisang Batu	*Musa*	*balbisiana*	type 4	Indonesia (IDN 080)
34	-	Schizocarpa	*Musa*	*schizocarpa*	-	Papua New Guinea

### DNA/RNA Extraction and cDNA Synthesis

Genomic DNA was extracted from frozen plant leaf tissue taken from single plants using the DNeasy Plant DNA Extraction kit (Tiangen, Beijing). To determine the structures of the *Musa Waxy* and *Adh*1 genes, total RNA was isolated from the unripe pulp of cultivar Haigong (AA genotype) using the RNAiso Plus and RNAiso-mate for Plant Tissue extraction kit (TaKaRa, Dalian). First-strand cDNA synthesis was performed using the PrimeScript™ 1st Strand cDNA Synthesis Kit (TaKaRa) following the manufacturer’s directions.

### Identification of *Musa* orthologs and sequence determination

The *Musa* orthologs of the *Waxy* and *Adh*1 genes were identified by performing a BLAST homology search [26] against the *Musa* EST database at NCBI using the *Oryza sativa* coding sequences as queries (GenBank accession numbers: *Waxy*: NM_001065985, *Adh*1: EF122490). The parameters for BLASTN (http://blast.ncbi.nlm.nih.gov/Blast.cgi) were set as follows: database was expressed sequence tags (est) and organism name was *Musa*. Sequences that were retrieved in this way were used to design primers with the software Primer Premier 5.0 (Premier Biosoft International, Palo Alto, CA). The positions of exons were determined by reference to the annotated *O. sativa* sequences. The homologs of *matK* and *trnL-F* in *Musa* accessions were amplified using published universal primer sequences [27], [28].

All polymerase chain reaction (PCR) amplifications were performed using a PTC-200 thermal cycler (MJ Research) in 30 µL volumes each containing the following components: 10–50 ng template DNA, 0.3 mM of each dNTP, 0.6 µM of each primer, 1×LA PCR buffer (Mg^2+^ plus), 1.5 unit of LA *Taq* polymerase (TaKaRa). Cycling parameters were: 94°C for 1 min, 30 cycles of (98°C, 10 s; 68°C, 2 to 6 min) and a final extension at 72°C for 10 min. All the amplified products were cloned into the pMD-18 vector (TaKaRa). To obtain all haplotypes for each *Musa* accession, between four and ten clones were sequenced per accession, and all sequences were determined using an ABI 3730 DNA analyzer (Applied Biosystems). Singleton variants were checked by sequencing multiple clones or from direct sequencing of PCR products.

### Data Analysis

Initial sequence editing and assembly were performed using ContigExpress (Informax Inc. 2000 North Bethesda, MD). DNA sequence alignment was implemented in ClustalX 1.83 [29] and if necessary alignments were edited manually in BioEdit 7.0.1 [30]. To infer the wild parents of cultivated bananas, the neighbor-joining (NJ) method for phylogenetic inference was carried out in MEGA version 5 [31], using Kimura’s 2- parameter distances [32]. Gaps were treated as missing data and bootstrap support values for the NJ trees were obtained from 1,000 replicates.

It has been proposed that AA diploid cultivated bananas were initially domesticated from wild relatives and triploid cultivars were then generated from diploid cultivars [2]. In addition, our phylogenetic analyses revealed that only the subspecies of *M. acuminata* that are distributed in the islands of Southeast Asia (ISEA) have donated genomes to domesticated bananas. We therefore estimated the overall nucleotide diversities of the *Waxy* and *Adh*1 genes in the A and B genomes of cultivated banana as well as in their respective wild relatives. We also evaluated the nucleotide diversity for AA, AAA, AAB, *M. acuminata* (island) and *M. acuminata* (mainland) groups separately. The nucleotide diversity of AS and ABB genotypes was not calculated because only one haplotype was obtained from these groups. Genetic analyses of sequence polymorphism were performed using DnaSP version 5 [33], values determined included number of segregating sites (S), number of haplotypes (H), Tajima’s D [34] and Fu and Li’s D* and F* [35]. In addition, we surveyed nucleotide diversity π [36] and θ [37] for total, silent and nonsynonymous sites independently; insertions/deletions (indels) were not included in this analysis. To further evaluate how domestication has modulated the genomic constitution of cultivated bananas, we estimated the allele frequencies of *Waxy* and *Adh*1 genes in both *M. acuminata* (island) and the A-genome of cultivated bananas using DnaSP version 5.

## Results

### Gene structures and primer sequences for *Waxy* and *Adh*1

To determine the exact structures of the *Waxy* and *Adh*1 genes in *Musa*, we sequenced cDNAs from the two single copy nuclear genes and predicted exon and intron boundaries by comparing them with the genomic sequences. Details of the *Waxy* and *Adh*1 gene structures and the primer sequences used are given in Table S1 and Figure S1. *Adh*1 gene sequences were obtained from all 34 *Musa* accessions and yielded a total of 61 unique haplotypes. The *Adh*1 gene contains eight exons and alignment of this gene produced 1,899 base pair (bp) matrix in length and 745 bp of which was located in exon. Similarly, a total of 56 unique haplotypes for the *Waxy* gene was obtained from the 34 *Musa* accessions. According to our analysis of gene structure, the *Waxy* gene in *Musa* contains of 14 exons and the aligned matrix was 4,469 bp in length, of which 1,809 bp were in coding regions. In addition, the two chloroplast genes (*matK* and *trnL-F*) generated 29 unique haplotypes and the alignment of the 34 accessions was 1,665 bp in length. All the DNA sequences obtained in this study have been submitted to GenBank under the accession numbers KC904543-KC904719.

### Genealogical patterns

**Figure 1:**
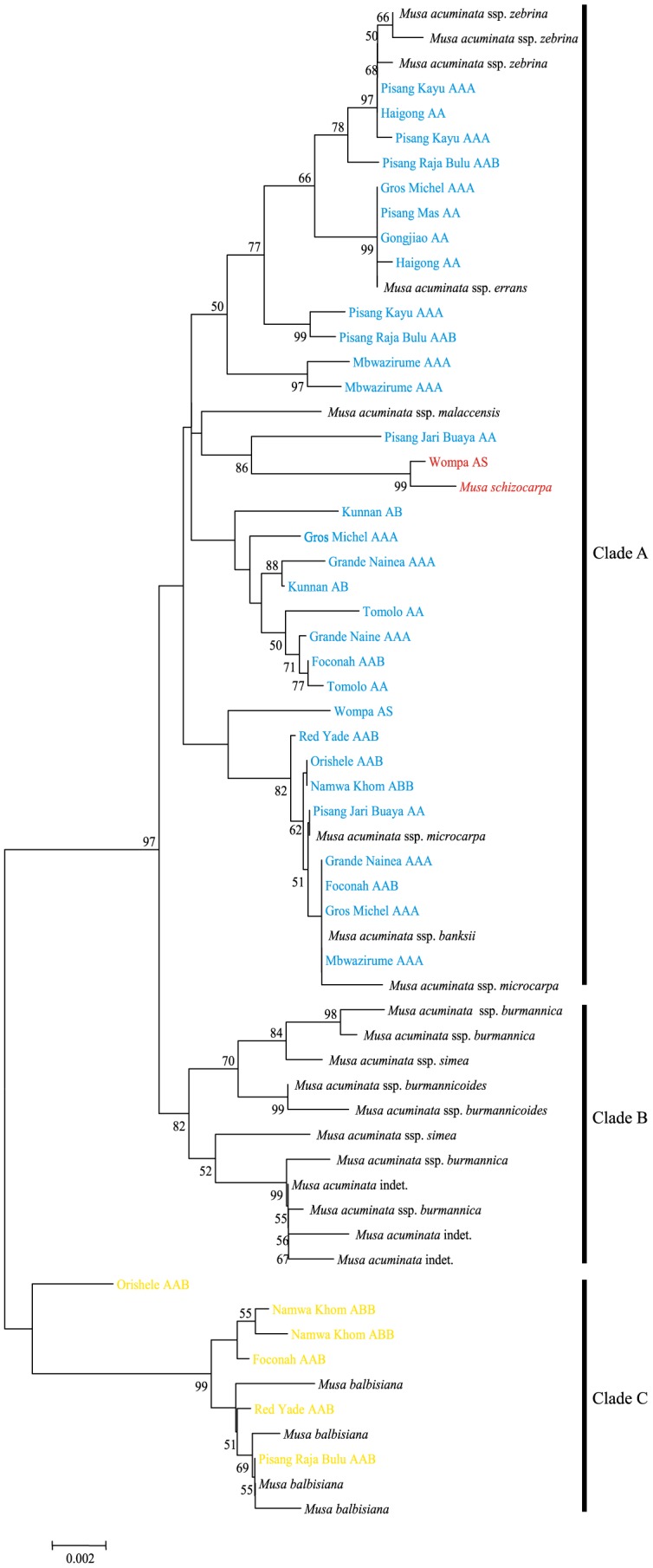
Neighbor-joining (NJ) tree for the *Adh1* gene from *Musa* accessions. Numbers are bootstrap percentages above 50%. Capital letters following each accession name indicate the previously-recognized genome composition of the cultivar. The appearance of an accession more than once represents distinct sequences cloned from the same cultivar. Color-coded names of accessions indicate the A (blue), S (red) and B (yellow) genomes of cultivated bananas and their wild relatives *M. acuminata* (black), *M. schizocarpa* (red) and *M. balbisiana* (black).

**Figure 2:**
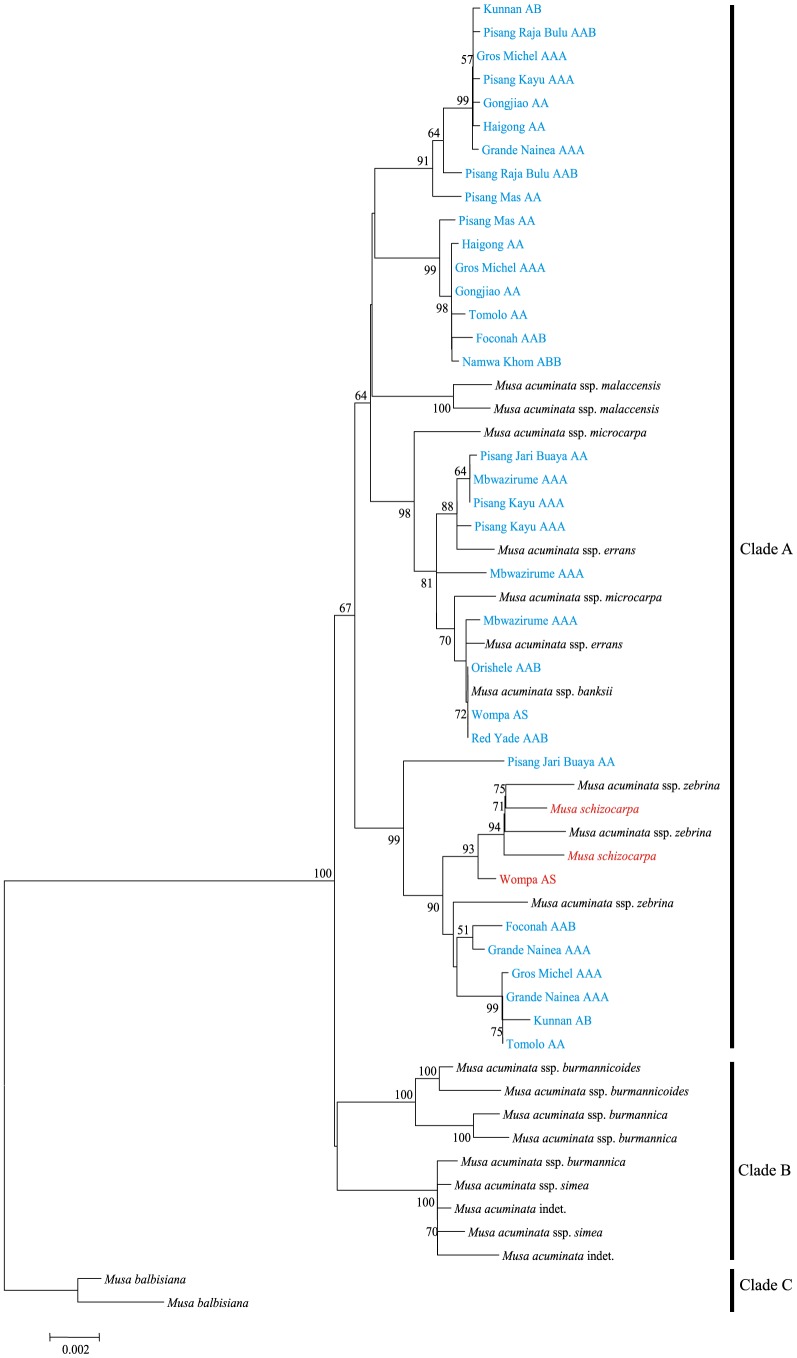
Neighbor-joining (NJ) tree for the *Waxy* gene in *Musa* accessions. Numbers are bootstrap percentages above 50%. Capital letters following each accession name indicate the previously-recognized genome composition of the cultivar. The appearance of an accession more than once represents distinct sequences cloned from the same cultivar. Color-coded names of accessions indicate the A (blue), S (red) and B (yellow) genomes of cultivated bananas and their wild relatives *M. acuminata* (black), *M. schizocarpa* (red) and *M. balbisiana* (black).

In order to address the systematic relationships among these *Musa* accessions, phylogenetic analyses were performed for *Waxy*, *Adh*1 and combined chloroplast DNA (cpDNA) datasets separately. Although some clades exhibited low bootstrap support (< 50%), the phylogenetic trees of both *Waxy* and *Adh*1 genes consisted of three major clades (Figures 1 and 2). As shown in Figure 1, in the case of *Adh*1, the basal clade (Clade C) contains the B genome haplotypes of Orishele (AAB), Namwa Khom (ABB), Foconah (AAB), Red Yade (AAB) and Pisang Raja Bulu (AAB) (yellow) and the wild species *M. balbisiana* (black). Clade B contains all of the haplotypes that were identified in the *M. acuminata* subspecies collected from the mainland of Southeast Asia (MSEA), including *burmannica*, *burmannicoides* and *siamea*. Finally, the remaining haplotypes were grouped together as a single clade (Clade A). It should be noted that, for clade A, the S genome haplotype of Wompa (AS) (red) clustered with its putative wild relative *M. schizocarpa*, and all the subspecies of *M. acuminata* in this clade are found only on the ISEA. These findings indicated that *M. balbisiana* and *M. schizocarpa* have indeed contributed to the gene pools of cultivated banana, and only the ISEA subspecies of *M. acuminata* have been involved in the process of domestication. Similar results were observed in the phylogenetic tree for the *Waxy* gene (Figure 2). Clade A consists of A-genome haplotypes of cultivated banana (shown in blue), ISEA subspecies of *M. acuminata* (black), the S genome haplotype of Wompa (red) and *M. schizocarpa*. Clade B includes all haplotypes of the MSEA subspecies of *M. acuminata*. For the Clade C, all of the B genome haplotypes failed to be amplified from five AAB and ABB genotype accessions and three *M. balbisiana* varieties (Honduras, Lal Velchi and Pisang Batu), suggesting that these three *M. balbisiana* varieties may have donated genomes to cultivated banana.

**Figure 3:**
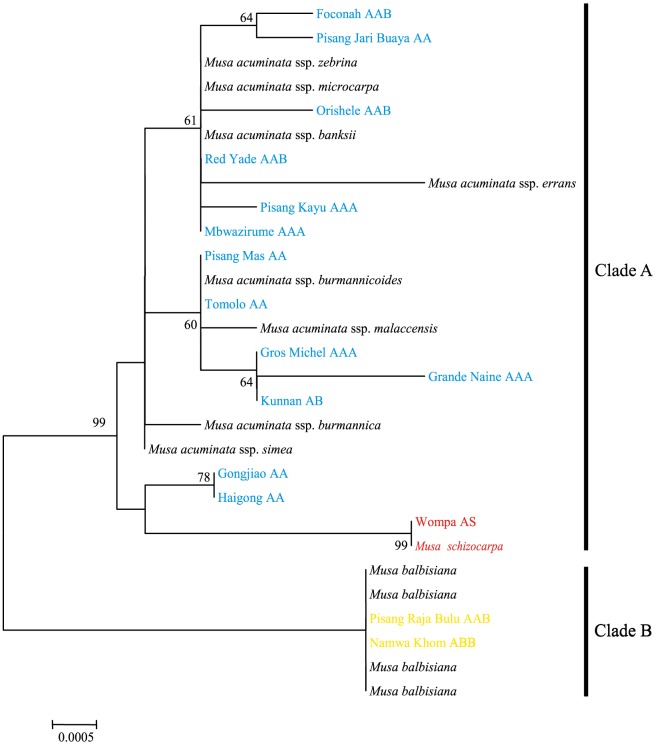
Neighbor-joining (NJ) tree inferred from the concatenated chloroplast (*matK* and *trnL-F*) fragments of *Musa* accessions. Numbers near branches are bootstrap percentages and branches without numbers indicate values lower than 50%. Capital letters following each accession name indicate the previously-recognized genome composition of the cultivar. Color-coded names of accessions indicate the A (blue), S (red) and B (yellow) genomes of cultivated bananas and their wild relatives *M. acuminata* (black), *M. schizocarpa* (red) and *M. balbisiana* (black).

We have also identified the putative paternal and maternal donors of each cultivated accession based on phylogenetic analyses of nuclear and cpDNA datasets (Figure 1, 2 and 3). For instance, the S genome haplotype of Wompa (AS) clustered together with *M. schizocarpa* in the cpDNA phylogenetic tree. In contrast, the A-genome haplotype of Wompa (AS) was grouped with *M. acuminata* ssp. *banksii*/*microcarpa*. This suggests that *M. schizocarpa* donated the maternal genome of Wompa (AS). Our analyses revealed that each genotypic group of cultivated banana originated from multiple intra- and inter-specific hybridization events. For example, five diploid AA cultivars from different points across the distribution range were investigated in this study. The results of phylogenetic analyses of nuclear and cpDNA markers revealed that Haigong and Gongjiao have similar maternal origins, and Pisang Mas and Tomolo may originate from the same maternal donor. Similar results were also observed in triploid AAB and ABB groups, in which different cultivars within the same group showed diverse paternal and maternal origins. Overall, our results not only confirmed the complex geodomestication process that has given rise to cultivated banana, but also shed further light on the multiple origins of each genotypic group.

### Nucleotide polymorphism and neutrality tests

DNA polymorphism analyses of the *Waxy* and *Adh*1 genes showed that genomic variations were abundant in these *Musa* accessions, with the total number of segregating sites being 167 in cultivated bananas (A genome) and 266 in *M. acuminata* (Table 2). In addition, 15 insertions/deletions (INDELs) were observed, all of them in introns of the two genes (data not shown). Two large INDELs were identified in *Waxy*, in introns 8 and 9.

**Table 2:** Summary of nucleotide diversity and neutrality test statistics for *Waxy* and *Adh*1 genes.

Gene	Taxonomic groups		Ha	S	π_T_	θ_T_	π_sil_	θ_sil_	π_non_	θ_non_	D	D*	F*
*Waxy*	A genome	AA	9	78	0.00741	0.00743	0.01117	0.01122	0.00056	0.00053	–0.01678	–0.31777	–0.27495
		AAA	11	81	0.00821	0.00696	0.01234	0.01040	0.00073	0.00072	0.83948	1.04499	1.13018
		AAB	5	74	0.00818	0.00830	0.01234	0.01253	0.00062	0.00063	–0.09452	–0.00493	–0.02642
		overall	26	102	0.00711	0.00570	0.01068	0.00846	0.00066	0.00073	0.94035	0.99006	1.15111
	B genome	overall	0	0	NA								
	*M. acuminata* (island)		9	112	0.00944	0.01064	0.01335	0.01466	0.00244	0.00345	–0.58381	–0.34486	–0.45283
	*M. acuminata* (mainland)		9	106	0.00910	0.00976	0.01233	0.01283	0.00302	0.00400	–0.35344	–0.64677	–0.64684
	*M. acuminata*		18	177	0.01041	0.01412	0.01485	0.01912	0.00304	0.00365	–1.11255	–1.18697	–1.35453
	*M. balbisiana*		2	22	NA								
*Adh*1	A genome	AA	7	52	0.01005	0.00977	0.01408	0.01348	0.00101	0.00144	–0.18205	–0.25666	–0.26684
		AAA	10	45	0.00524	0.00440	0.01425	0.01209	0.00000	0.00000	0.81799	1.04267	1.12131
		AAB	6	41	0.00965	0.00911	0.01396	0.01319	0.00000	0.00000	–0.06741	–0.15142	–0.14622
		overall	23	65	0.00931	0.00775	0.01342	0.01086	0.00024	0.00089	0.08433	–0.73278	–0.54624
	B genome	overall	6	34	0.00712	0.00811	0.01031	0.01175	0.00000	0.00000	–0.77848	–0.88204	–0.94087
	*M. acuminata* (island)		8	48	0.01190	0.01166	0.01595	0.01476	0.00279	0.00469	0.34526	0.26549	0.31497
	*M. acuminata* (mainland)		11	46	0.00814	0.00853	0.01119	0.01126	0.00129	0.00242	–0.32476	–0.95142	–0.89668
	*M. acuminata*		19	89	0.01178	0.01393	0.01642	0.01861	0.00147	0.00354	–0.63949	–0.79230	–0.86965
	*M. balbisiana*		4	12	0.00324	0.00354	0.00351	0.00383	0.00265	0.00289	–0.84046	–0.84046	–0.84986

Ha: number of haplotypes.

S: number of segregating sites.

π_T_: average number of nucleotide differences per site between two sequences (Nei, 1987) calculated for the total number of polymorphic sites.

π_sil_: average number of pairwise nucleotide differences per site calculated for the silent sites.

π_non_: average number of pairwise nucleotide differences per site calculated for the nonsynonymous sites.

θ_T_: Watterson's estimator of θ per base pair (Watterson, 1975) calculated for the total number of polymorphic sites.

θ_sil_: Watterson’s estimator of θ per base pair calculated for the silent sites.

θ_non_: Watterson’s estimator of θ per base pair calculated for the nonsynonymous sites.

D: Tajima’s D (Tajima 1989).

D*/F*: Fu & Li’s D*, Fu & Li’s F* (Fu and Li 1993).

Estimates of nucleotide diversity (π and θ) for all cultivated and wild *Musa* accessions were performed for silent, nonsynonymous and total sites independently. Summaries of nucleotide diversity data for the two nuclear genes are given in Table 2. Reduced levels of polymorphism emerged as a general property of cultivated bananas relative to their wild progenitors. For example, the nucleotide diversity values (π and θ) of the *Waxy* and *Adh*1 genes demonstrated that *M. acuminata* has slightly higher nucleotide diversity than the A-genome of cultivated bananas at total and silent sites (Table 2). As the phylogenetic trees had revealed that only the ISEA subspecies of *M. acuminata* have donated genomes to cultivated banana, we divided the *M. acuminata* accessions into an island and a mainland group. Results from the analysis of DNA polymorphisms showed that diploid AA cultivars harbored only slightly lower nucleotide diversity than that of *M. acuminata* (island) (Table 2). These findings suggested that the cultivated banana may not have undergone any very severe genetic bottleneck during the initial domestication process. Similarly, both the triploid AAA and AAB groups also possessed high levels of nucleotide diversity, indicating that the historical population size of the triploid bananas may also have been large. The B-genome of cultivated banana showed higher nucleotide diversity than that of *M. balbisiana* (Table 2).

**Figure 4:**
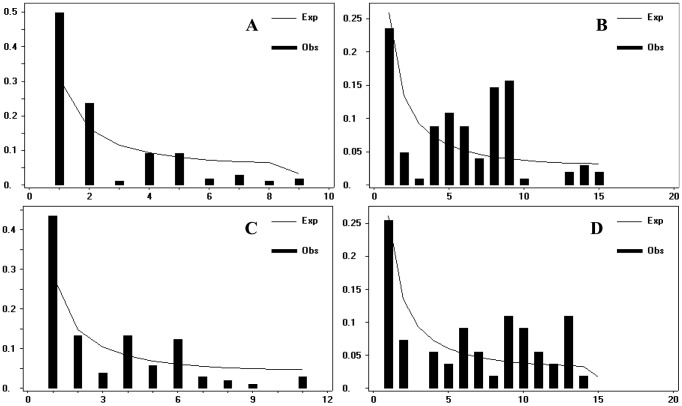
Allelic frequency of the *Waxy* and *Adh*1 genes. (A) *Waxy* gene for all *M. acuminata* **accessions**; B: *Waxy* gene for **the** A genome of cultivated banana; C: *Adh*1 gene (*M. acuminata*); D: *Adh*1 gene (cultivated banana).

Interestingly, we found that nucleotide diversity at nonsynonymous sites in both *Adh*1 and *Waxy* genes was reduced in the A-genome as well as within each cultivar genotype. No polymorphic sites in the *Adh1* gene were observed within the AAA and AAB groups. Theoretically, reduced genetic diversity at nonsynonymous sites usually implies that artificial selection may have acted on the coding regions. Nonetheless, it was found that although the genetic diversity of *M. acuminata* was 4- to 6-fold higher than those of A-genome cultivars, about half the numbers of nonsynonymous mutations were identified in the A-genome of cultivated bananas (4 and 23 mutations for *Adh*1 and *Waxy*, respectively) compared with *M. acuminata* (8 and 44 mutations for *Adh*1 and *Waxy*, respectively). Additionally, the patterns of nucleotide variations in *Waxy* and *Adh*1 were examined for deviation from neutral equilibrium evolution using the Tajima’s D and Fu and Li’s D* and F* tests. As expected, no significant departures from the neutral model were observed in any test. This observation was further examined by an analysis of allele frequencies, in which cultivated bananas showed an excess of intermediate frequency variants in both *Waxy* and *Adh*1 (Figure 4).

## Discussion

Domestication is a complex evolutionary process in which human use of plants and animals result in phenotypic changes that distinguish domesticated species from their wild progenitors [38], [39]. In recent decades, many researchers have conducted studies of how, and to what extent, different plants and animals were domesticated from wild relatives [40], [41], [42], [43], [44], [45], [46]. Here, we address the origin and domestication of cultivated bananas using results obtained with two single copy nuclear (*Waxy* and *Adh*1) and two chloroplast (*mat*K and *trnL-F*) genes.

### Origin and domestication of cultivated bananas

It has been suggested that AA diploid cultivars were directly domesticated from *M. acuminata* [1], [2], [47], [48]. To further address the origin of, and the domestication process that gave rise to, cultivated bananas, five diploid AA accessions and seven *M. acuminata* subspecies representing their geographical ranges were investigated. Results from phylogenetic analyses revealed that diploid AA cultivars were initially domesticated from ISEA through multiple intra-specific hybridizations between different subspecies of *M. acuminata*. This finding confirmed the previous hypothesis that cultivated bananas originated from three contact zones in ISEA through complex geodomestication pathways [2]. Perrier et al. [2] have proposed that the subsp. *banksii* and *errans* contributed to most of the diploid AA cultivars. However, our results demonstrated that the subsp. *malaccensis* might contribute genetically to most of the five AA cultivars. In addition, we have shown that the five AA cultivars investigated here have diverse maternal donors, suggesting that multiple maternal origins may be a general characteristic of the AA cultivars.

The origin of AAA triploid bananas has long been debated, and the most accepted view holds that they are derived from hybridizations between AA diploid cultivars [2], [49], [50], [51]. In the present study, four AAA triploid accessions collected from Southeast Asia, Africa and American were evaluated, and the phylogenetic analyses generated similar genealogical patterns to those of AA cultivars. These results further confirmed a previous hypothesis that triploidization may occur separately in various geographic regions through multiple hybridizations between different diploid AA cultivars and subsequent spread to other regions of the world [1], [2]. It is noteworthy that previous studies demonstrated that the cultivars Gros Michel (AAA) and Grande Naine (AAA), which comprise over 50% of banana production, have a common diploid ancestor [52], [53]. Our study has further revealed that subsp. *malaccensis*-derived diploid AA cultivars may have donated maternal genomes to these cultivars.

It has been proposed that *M. balbisiana* and *M. schizocarpa* have also contributed to the gene pools of modern bananas [1], [2]. In this study, seven cultivated accessions belonging to the AB, AS, AAB and ABB genotype groups were investigated. According to the phylogenetic trees (Figure 1, 2 and 3), at least one haplotype of each of these accessions was clustered together with *M. balbisiana* (shown in yellow) and *M. schizocarpa* (red), with the exception of Kunnan (AB genotype), in which all of the haplotypes grouped together with *M. acuminata* (blue). Our findings here allow us to propose that *M. balbisiana* and *M. schizocarpa* have indeed donated B and S genomes to domesticated banana, and that the genotype of Kunnan should be denoted AA. It has been suggested that the B genome of cultivated banana may originate from southern China [2]. In this study, our results demonstrated that only two *M. balbisiana* (Pisang Klutuk Wulung and Tani) accessions gave successful amplification of the *Waxy* gene, suggesting these accessions may have had no involvement in the process of domestication of cultivated banana. In addition, our results revealed that *M. schizocarpa* donated maternal genomes to the cultivar Wompa (AS). Similarly, although five AAB and ABB accessions contained B genomes, *M. balbisiana* donated maternal genomes only to Pisang Raja Bulu (AAB) and Namwakhom (ABB). These findings suggest multiple inter-specific hybridization origins for the B genome in domesticated bananas.

Overall, our study has not only confirmed previous findings that cultivated banana was initially domesticated from ISEA through complex geodomestication pathways, but also demonstrated that multiple maternal origins may be a general phenomenon in all diploid and triploid cultivars.

### Genetic diversity of *Musa* germplasm

Theoretically, cultivated plants are usually expected to undergo severe genetic bottleneck events during the initial domestication and subsequent improvement processes, and such events may result in a sharp reduction in genetic diversity [54], [55]. This hypothesis has been well documented in genetic studies of rice (*Oryza sativa*), wheat (*Triticum aestivum*) and other crops [56], [57], [58]. To evaluate the germplasm of domesticated bananas, several studies have investigated the genetic diversity of cultivated and wild *Musa* accessions using isozyme [59], [60], AFLP [14], [15], microsatellite [18] and PCR-RFLP [61] markers. These studies have demonstrated that domesticated bananas harbor high levels of genetic diversity.

In this study, we evaluated the nucleotide diversity of 34 wild and cultivated *Musa* accessions by assessing the DNA polymorphisms of the *Waxy* and *Adh*1 genes. As expected, although only five diploid AA accessions were investigated, slightly lower nucleotide diversity was observed in *Waxy* and *Adh1* genes in these accessions relative to their wild parent *M. acuminata* (island). High nucleotide diversity in cultivated banana implies that it may have had a historically large population size and did not undergo any severe genetic bottleneck during the domestication process. Nonetheless, high degrees of phenotypic divergence between different cultivars were observed, suggesting that artificial selection may have acted on morphological traits in such a way as to decrease the genetic diversity of domesticated bananas. Two factors may explain this phenomenon: the first is that multiple intra-specific hybridizations between subspecies of *M. acuminata* led repeatedly to the occurrence of mutant lines with seedless and parthenocarpic fruit, and this may have brought about an increase in the number of initial founders of domesticated bananas. This hypothesis has been put forward previously, and our present study shows that cultivated bananas have arisen through complex geodomestication pathways [1], [2]. This allows us to speculate that multiple hybridization origins may be at least partly responsible for the enormous nucleotide diversity of diploid AA cultivars. Multiple morphological variants were also found among the subsp. of *M. acuminata* [4], [62], and these may have contributed to the phenotypic variation found in domesticated banana. Secondly, most cultivars were initially collected from the wild by farmers and then brought into cultivation via vegetative propagation. This will have facilitated preservation of selected somatic clonal variants with useful traits, such as inflorescence, fruit and height characteristics [63], [64], [65]. Taken together, accumulation of mutations resulting from hybridization as well as new somatic mutations may be responsible for the high nucleotide diversity of AA cultivars. Triploid cultivars (e.g., AAA, AAB and ABB) originated from diploid AA cultivars, and high levels of nucleotide diversity were also found in the A and B genomes (Table 2). As shown in the phylogenetic analyses (Figures 1, 2 and 3), triploid cultivars (including AAA and AAB) have also undergone multiple hybridization events that may have resulted in the observed high levels of nucleotide diversity.

It should be noted that nucleotide diversity at nonsynonymous sites in the *Waxy* and *Adh*1 genes was reduced in cultivated banana in comparison with its wild relatives, implying that functional constraints may affect the coding regions. It has been shown that the *Waxy* gene plays a crucial role in amylose synthesis [19]. Several *Waxy* mutants (null alleles) have been identified in maize, rice and barley, and these null alleles down-regulate amylase expression. Thus, *Waxy* (amylose free or glutinous) mutants became targets of artificial selection during domestication and subsequent crop improvement processes [66], [67]. In domesticated banana, however, although the amylose content in total reserve starch varies in different cultivars (from 15% to 25%), the percentages of amylose in most cultivars are usually below 19% [68]. In addition, our study showed that no *Waxy* mutants were found in these *Musa* accessions and there were no significant departures from the neutral model in any statistical tests. These observations allow us to infer that artificial selection may have not acted on the *Waxy* gene of cultivated bananas. This phenomenon was also observed in the *Adh*1 gene, in which nucleotide diversity at nonsynonymous sites decreased in cultivars relative to wild accessions. The *Adh*1 gene, a member of the alcohol dehydrogenase gene family, has been shown to be a neutral locus in grass crops [57], [69]. Only a small number of nonsynonymous mutations were identified in the A-genomes of cultivated bananas and of the ISEA subspecies of *M. acuminata*. Therefore, an overall decrease in the number of mutations might have affected the nucleotide diversity at nonsynonymous sites. Overall, our findings implied that the *Waxy* and *Adh*1 genes may not have been under artificial selection during the domestication process. However, this finding requires further investigation in order to address the question of whether a reduction in nucleotide diversity at nonsynonymous sites is a general attribute in cultivated bananas.

## Conclusion

Previous studies in the fields of archaeology, genetics and linguistics have shed light upon the major stages in the domestication process of cultivated bananas [1], [2], [70]. In this study, we have not only confirmed the multiple intra- and inter-specific hybridization origins of cultivated banana, but also revealed that both diploid and triploid cultivars harbor high levels of nucleotide diversity. However, only a small number of *Musa* accessions were investigated in this study, and it may therefore be insufficiently representative of the genus. It will be necessary to employ tens of nuclear genes and hundreds of *Musa* accessions in future studies to further elucidate the domestication process undergone by cultivated banana.

## Supporting Information

Figure S1 **Structures of** ***Waxy*** **and** ***Adh*** **1 genes in** ***Musa*** **and the positions of each primer used in this study.**
(EPS)

Table S1 **The sequences of primers and the information of Waxy and Adh1 gene used in this study.**
(DOCX)
