# Supplementary material for: Origins and Domestication of Cultivated Banana Inferred from Chloroplast and Nuclear Genes
Source: PLoS One. 2013 Nov 18;8(11):e80502. doi: 10.1371/journal.pone.0080502 (PMC3832372; doi:10.1371/journal.pone.0080502)
Supplement: Table S1 — The sequences of primers and the information of Waxy and Adh1 gene used in this study. (DOCX) [file pone.0080502.s002.docx]

**Table S1** The sequences of primers and the information of *Waxy* and *Adh1* gene used in this study.

| Locus | Alignment length (bp) | | | Primer sequence (5’-3’) | Functional association |
| --- | --- | --- | --- | --- | --- |
|  | Total | Exon | Intron |  |  |
| *Waxy* | 4469 | 1809 | 2660 | WPF: 5- ATTCTCAATGGCTGCTGTAAC-3 | ground-bound starch synthase |
|  |  |  |  | WPR:5-CAGGAGGATCAAACTCAAACAC-3 |  |
|  |  |  |  | WSA: 5- ATGGCTGCAAATGGACAC-3 |  |
|  |  |  |  | WSB: 5-TCCTTATTCAGAGGCTTTGC-3 |  |
|  |  |  |  | WSC: 5- GAATGGGATGGACACTAACG-3 |  |
|  |  |  |  | WSD: 5- TGAAAACCCTGTCAACTCCT-3 |  |
|  |  |  |  | WSE: 5-GTATATCCCGTGAGATTGGTA-3 |  |
| *Adh1* | 1899 | 754 | 1145 | APF: 5- ACACCGATGTCTACTTCTGG-3 | alcohol dehydrogenase |
|  |  |  |  | APR: 5- CTGTACGTGGCTTATAGTTCC-3 |  |
|  |  |  |  | ASA: 5- CGACAGAGGAGTGATGATAG-3 |  |
